# The claudin gene family: expression in normal and neoplastic tissues

**DOI:** 10.1186/1471-2407-6-186

**Published:** 2006-07-12

**Authors:** Kyle J Hewitt, Rachana Agarwal, Patrice J Morin

**Affiliations:** 1 Laboratory of Cellular and Molecular Biology, National Institute on Aging, Baltimore MD 21224, USA; 2 Department of Pathology, Johns Hopkins Medical Institutions, Baltimore, MD 21287, USA

## Abstract

**Background:**

The claudin (*CLDN*) genes encode a family of proteins important in tight junction formation and function. Recently, it has become apparent that *CLDN *gene expression is frequently altered in several human cancers. However, the exact patterns of *CLDN *expression in various cancers is unknown, as only a limited number of *CLDN *genes have been investigated in a few tumors.

**Methods:**

We identified all the human *CLDN *genes from Genbank and we used the large public SAGE database to ascertain the gene expression of all 21 *CLDN *in 266 normal and neoplastic tissues. Using real-time RT-PCR, we also surveyed a subset of 13 *CLDN *genes in 24 normal and 24 neoplastic tissues.

**Results:**

We show that claudins represent a family of highly related proteins, with claudin-16, and -23 being the most different from the others. From *in silico *analysis and RT-PCR data, we find that most claudin genes appear decreased in cancer, while *CLDN3*, *CLDN4*, and *CLDN7 *are elevated in several malignancies such as those originating from the pancreas, bladder, thyroid, fallopian tubes, ovary, stomach, colon, breast, uterus, and the prostate. Interestingly, *CLDN5 *is highly expressed in vascular endothelial cells, providing a possible target for antiangiogenic therapy. *CLDN18 *might represent a biomarker for gastric cancer.

**Conclusion:**

Our study confirms previously known *CLDN *gene expression patterns and identifies new ones, which may have applications in the detection, prognosis and therapy of several human cancers. In particular we identify several malignancies that express *CLDN3 *and *CLDN4*. These cancers may represent ideal candidates for a novel therapy being developed based on CPE, a toxin that specifically binds claudin-3 and claudin-4.

## Background

The claudin family consists of approximately 23 proteins that are essential for the formation of tight junctions (TJs) in epithelial and endothelial cells [1]. TJs have crucial roles in the control of paracellular transport and in the maintenance of cell polarity. It is thought that various claudin family members can confer different properties to epithelial cell permeability and account for some of the selective variability of different barriers [1]. Indeed, most tissues express multiple claudins, which can interact in both homotypic and heterotypic fashion to form the tight junction strands. The exact combination of claudin proteins within a given tissue is thought to determine the selectivity and strength of the tight junctions. Underscoring the critical roles of claudin proteins are recent observations that germline mutation in these genes can lead to various familial diseases, such as neonatal sclerosing cholangitis (*CLDN1*) [2], nonsyndromic recessive deafness (*CLDN14*) [3], and familial hypomagnesaemia (*CLDN16*)[4].

Recent gene expression profiling analyses have shown that claudin gene expression is frequently altered in various cancers (reviewed in [5,6]). For example, *CLDN3*, and *CLDN4 *have been found frequently up-regulated in ovarian, breast, prostate and pancreatic tumors [7-11]. *CLDN7 *has been found downregulated in breast and head and neck cancer, but elevated in stomach cancer [12,13]. *CLDN1 *is typically downregulated in various cancers, but has also been reported to be elevated. The picture that emerges suggests that claudin expression is altered in several human tumors. Specifically, *CLDN1,3,4,5,7,10,16 *have been found altered in various cancers [5]. The overexpression of these proteins in cancer (which typically lose their TJs) is unexpected but may be related to roles that are unrelated to TJ formation [5]. Indeed, recent work suggests that claudins may be involved in survival and invasion of cancer cells [12,14,15].

Regardless of their exact functions in cancer cells, claudin protein expression may have significant clinical relevance [5,6]. For example, claudin-1 expression has been shown to have prognostic value in colon cancer [16], claudin-18 in gastric cancer [17], and claudin-10 in hepatocellular carcinoma [18]. In addition, because claudins are surface proteins, they may represent useful target for various therapeutic strategies. Of particular interest, in the possible use of *Clostridium perfringens *enterotoxin (CPE) as a novel chemotherapeutic compound. CPE is a natural ligand for claudin-3 and -4 proteins, and binding of the toxin to these claudins leads to a rapid cytolysis of the cells [19]. Recent preclinical studies have suggested that CPE may be effective against claudin-3 and -4-expressing malignancies [8,9,11,20].

Unfortunately, the exact patterns of expression of the various claudins in different cancers and normal tissues are not well known. To date, only a few of the claudin proteins have been investigated in a relatively limited number of cancers. In this report, we use the vast amount of data present in the public SAGE database to create a claudin gene expression profile of all the known claudin genes, in a large number of tissues. We then survey a subset of these claudin genes using real-time RT-PCR in a panel of normal and neoplastic tissues. Our study confirms previous claudin gene expression patterns and identifies new ones, which may potentially be of clinical use for various cancers.

## Methods

### Claudin homology and phylogenetic tree

21 human claudin genes and corresponding proteins sequences were identified and downloaded from GenBank. The ClustalW software (with the Blosum62 matrix) was used to produce a multiple sequence alignment of all these human claudin protein sequences and the Jalview software was then used to visualize the results [21]. A phylogenetic tree of the claudin proteins was produced with ClustalW. The clustalW phylogenetic calculations are based on the neighbor-joining method of Saitou and Nei [22].

### In silico analysis of claudin gene expression

Mining of the SAGE Genie database [23] for libraries that expressed *GAPDH *or *ACTB *yielded a total of 266 SAGE libraries with at least some level of expression of these control genes. These libraries were then examined for the expression of all 21 human *CLDN *genes that we identified. SAGE data for both normal and cancerous tissues was exported to an excel spreadsheet, and expression levels converted to tags per 200,000 (the complete dataset is available as additional file 1). The dChip software  program was then used to visualize this data, assigning darker shades of red to higher number of tags.

### Real-time RT-PCR of claudin family members

A total of 48 cDNA preparations from various normal and neoplastic tissues (24 each) were purchased from Biochains (Hayward, CA). The GeneAmp 7300 Sequence Detection System (PE Applied Biosystems) was used for detecting RT-PCR products in real-time with the SYBR Green I assay, as previously described [24]. The primers for the various *CLDN *genes (*CLDN1,2,3,4,5,7,8,9,10,11,12,16,18*) and the control *GAPDH *were designed to cross intron-exon boundaries to distinguish PCR products generated from genomic versus cDNA template. For *CLDN *genes lacking introns, real-time RT-PCR was performed by the polyA cDNA-specific RT-PCR method [25]. The primer sequences are available online as additional file 2.

Each PCR reaction was optimized to ensure that a single band of the appropriate size was amplified and that no bands corresponding to genomic DNA amplification or primer-dimer pairs were present. The PCR cycling conditions were performed for all samples as follows: 50°C, 2 minutes for AmpErase UNG incubation, 95°C, 10 minutes for AmpliTaq Gold activation, and 40 cycles for the melting (95°C, 15 seconds) and annealing/extension (60°C for 1 minute) steps. PCR reactions for each template were done in duplicate in 96-well plates.

The comparative C_T _method (PE Applied Biosystems, Foster City, CA) was used to determine relative quantitation of gene expression for each *CLDN *gene compared to the *GAPDH *control. First, the C_T _values from *GAPDH *reactions were averaged for each duplicate. Next, the relative difference between *GAPDH *and each duplicate was calculated as previously described [24]. The final values were then averaged for each duplicate set, and used in the dChip analysis. Clustering of the *CLDN *genes was performed with distances based on 1-rank correlation and the centroid linkage method.

## Results

### The claudin family of proteins

**Figure 1:**
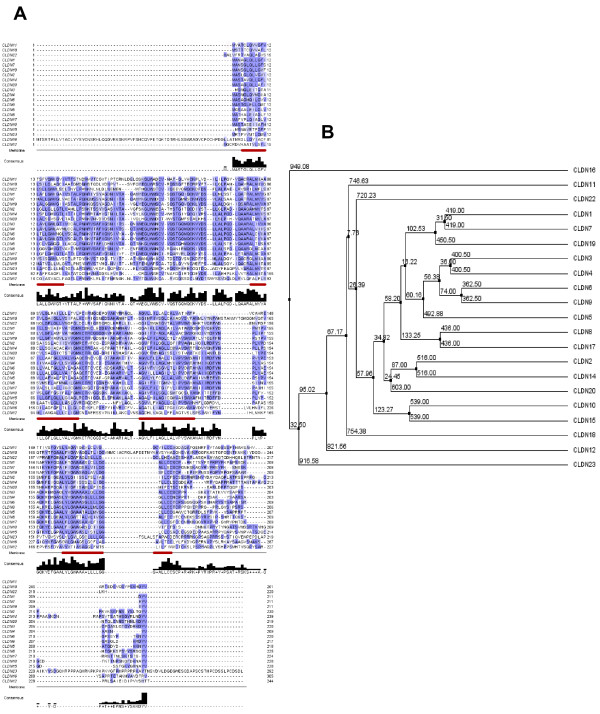
Multiple alignment and family tree of claudin proteins. A. ClustalW was used to generate alignment of all the human claudin protein sequences and the residues were then colored using Jalview [21] according to amino acid conservation among family members. Red bars underneath alignment indicate predicted transmembrane domains. The level of conservation and the consensus are also shown below the alignment. B. Phylogenetic tree of claudin family members. The tree was generated using ClustalW and visualize in Jalview. The numbers on the branches are Blosum62 scores, indicating distances among family members.

As a starting point for our analyses, we identified 21 different human claudin proteins in the GenBank database. Alignment of these 21 sequences using ClustalW shows that most of the claudin proteins are extremely similar, especially in the membrane-spanning regions (Figure 1A). Notable exceptions are claudin-16, which contains a 66 aa extension at the N-terminus, claudin-18, which has an extension in the second extracellular loop, and claudin-23 with a longer C-terminal tail. A phylogenetic tree was also generated to better identify similar members of the family. Overall, the tree demonstrated that the claudins constitute highly related family of proteins, with claudin-16, and -23 being the most different from the others (Figure 1B). Claudin-6 and -9 are the most similar, followed by claudin-3 and -4, and claudin-1 and -7.

### In silico analysis of CLDN expression in 266 tissues

**Figure 2:**
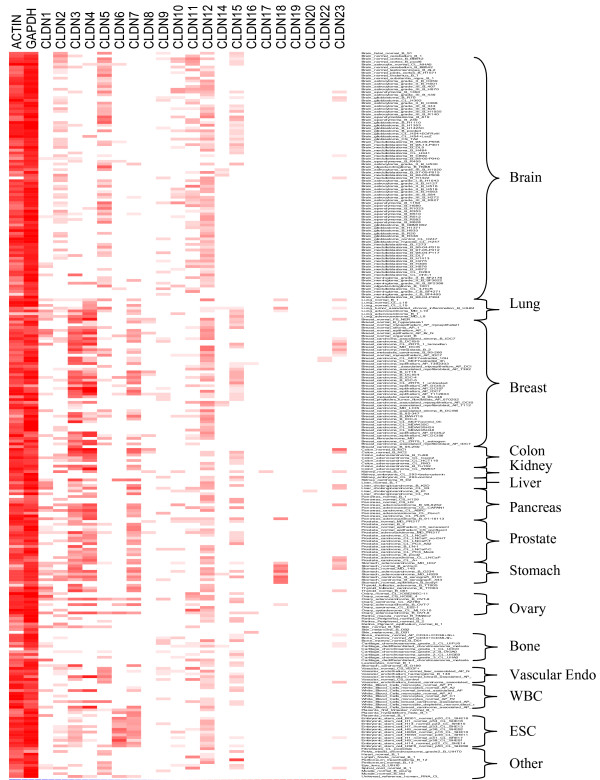
*In silico *analysis of claudin gene expression in various normal and neoplastic tissues. 266 SAGE libraries were examined for the expression of all 21 *CLDN *genes using SAGE Genie [23]. SAGE data was compiled from both normal and cancerous tissue, and analyzed using dChip software  program, which assigned darker shades of red to higher number of tags. GAPDH and ACTN levels were also analyzed as controls. While some *CLDN *genes are ubiquitously expressed, others exhibit highly tissue specific patterns. A detailed spreadsheet of the data is available as supplementary information.

Because serial analysis of gene expression (SAGE) measures absolute levels of transcripts, cross-comparison of SAGE data is possible across experiments and laboratories [26]. The SAGE Genie database has been developed to allow *in silico *analysis of gene expression and comparison of transcript levels in a large number of normal and diseased tissues [23]. Using the SAGE Genie database, we extracted gene expression data for all 21 human claudins across 266 tissues (Figure 2 and additional file 1). Some of these genes, such as *CLDN1,2,3,4,5,7,11,12*, and *15 *are expressed in a large number of different tissues. In contrast, other claudins, such as *CLDN14,16,17,20*, and *22 *have much more restricted expression patterns. *CLDN17*, for example, was found expressed in only one SAGE library (normal kidney), at low levels. *CLDN20 *was only found in 3 cancer libraries total (a chondrosarcoma, a brain cancer, and a liver tumor). Similarly, *CLDN22 *was only found in 2 breast cancer libraries and one brain astrocytoma library. On the other hand, *CLDN3,4*, and *7 *were highly expressed in most normal epithelial cells as well as their corresponding neoplasias. Further analysis suggested that these claudins are frequently elevated in cancer. For example, *CLDN3 *was elevated in tumors of the lung, prostate, breast, kidney, and ovary compared to their normal counterparts. Similarly, *CLDN4 *was elevated in tumors of the lung, breast, stomach, pancreas, and ovary. *CLDN7 *was elevated in cancers of the thyroid, lung, stomach, pancreas, liver, kidney, and ovary. Interestingly, *CLDN6 *was frequently expressed in embryonic stem cells (ESCs) but generally not in other tissues. *CLDN7 *was the only other *CLDN *expressed at significant level in ESCs. *CLDN18 *expression seemed to be mostly restricted to the stomach and the lungs. Vascular endothelial cells expressed *CLDN5 *at high levels, suggesting a new target for antiangiogenic therapy. Brain had distinctive claudin expression profiles, with *CLDN3*,*4 *and 7, expressed at low levels but *CLDN2 *and *CLDN5 *very highly expressed. This pattern was opposite to what we observed in epithelial cells. *CLDN12 *was the most widely expressed gene and appeared expressed constitutively in most tissues.

### Real-time RT-PCR analysis of CLDN expression

**Figure 3:**
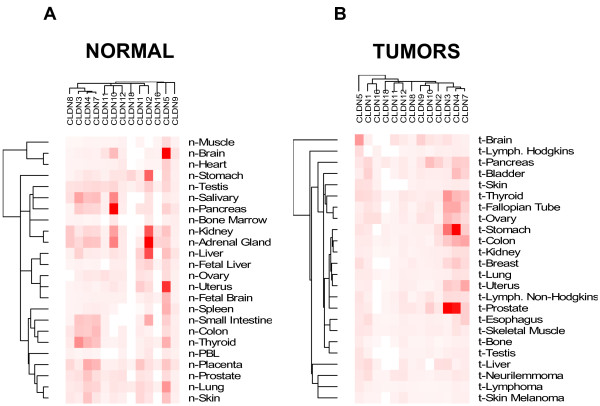
Real-time RT-PCR survey of *CLDN *gene expression in normal and neoplastic tissus. Expression data of 13 selected *CLDN *family members from 24 normal and 24 tumor tissue cDNAs. The dChip software was used to analyze and cluster the data. Clustering of the *CLDN *genes and the tissues were performed with distances based on 1-rank correlation and the centroid linkage method. Darker shades of red indicate higher *CLDN *expression.

In order to validate and extend the *in silico *results obtained with the SAGE Genie database, we performed real-time RT-PCR analysis on a subset of *CLDN *genes (13 genes total) to survey gene expression in several tissues. Gene-specific primers for *CLDN1,2,3,4,5,7,8,9,10,11,12,16,18 *were designed and optimized. These *CLDN *genes were chosen because they were expressed at detectable levels in several tissues and represented a wide variety of different expression patterns as suggested by the SAGE database analysis. A total of 24 normal tissues and 24 tumors were surveyed for *CLDN *expression (Figure 3). Using this technique, we find that the various normal tissues express a wide variety of *CLDN *genes (Figure 3A). For example, the kidney expresses high levels of *CLDN10 *and *CLDN12*, but also expresses some levels of all the other *CLDN *genes tested (with the exception of *CLDN18*). Clustering of *CDLN *genes showed a tight association of *CLDN3,4,7 *in terms of their expression patterns, suggesting a coordinate regulation of these genes. The *CLDN3,4,7 *cluster was found expressed at high levels in normal pancreas, salivary gland, kidney, adrenal gland, small intestine, colon, and thyroid. Examination of the tumor samples revealed that the diversity of *CLDN *expression was decreased in these samples (Figure 3B). Except for the *CLDN3,4,7 *cluster, which was also present and often elevated in tumors, the other *CLDN*s appeared to be expressed at relatively low levels. *CLDN3,4,7 *were expressed in tumors of the pancreas, bladder, thyroid, fallopian tubes, ovary, stomach, colon, breast, uterus, and prostate. Because of the low number of samples examined, this survey doe not represent an exhaustive analysis of *CLDN *gene expression in various tissues but rather an initial study of tissue specificity. However, the similarity in the gene expression patterns identified through this survey and previously known patterns (for *CLDN3 *and *4*, for example) is striking. Additional studies with several tumors, including various subtypes, grades, stages, and other clinical parameters will be necessary.

## Discussion

Alterations in the expression levels of tight junction proteins, especially claudins, continue to be reported in several cancers. However, an overall view of claudin gene expression in normal and cancer tissues has been lacking. In this report, we first use the large public SAGE database to investigate claudin expression of the 21 human *CLDN *genes we have identified in GenBank. We find that, while some *CLDN *genes are ubiquitously expressed, the majority of these genes exhibit a very restricted expression pattern. *CLDN14,16,17,19,20*, and *22*, for example, are found in only a few rare libraries. Others such as *CLDN3,4,5,7,11*, and *12 *are much more widely expressed. Our analysis allows for the identification of general expression patterns, such as the high expression of *CLDN3,4 *and *7 *in epithelial tissues, and lower expression in other tissues, such as the brain.

Our data also reveal claudin expression patterns that were not previously known and that may have clinical implications. According to our data, gastric cells (both normal and neoplastic) express high levels of *CLDN18*, while other tissues do not express this gene. Interestingly, a recent study shows that claudin-18 is highly expressed in normal gastric cells and that this high expression is retained in approximately half the gastric tumors [17]. Because of its highly restricted pattern, claudin-18 may therefore represent a useful target for therapy of gastric cancer, especially in those tumors that maintain high levels of this gene. Claudin-18 is likely involved in TJ formation in normal gastric cells, while cancer cells, which typically do not form TJ's, may have a more available form of claudin-18. Therefore cancer cells may be more sensitive to therapy involving the targeting of this molecule. We also find that *CLDN5 *is not generally expressed in epithelial tissues but is expressed at high levels in all vascular endothelial cell libraries analyzed. Although also expressed in the brain, *CLDN5 *may represent a target for antiangiogenic therapy, especially if using compounds that cannot cross the blood-brain barrier.

Our RT-PCR experiments provide a more quantitative look at claudin gene expression in several normal and neoplastic tissues. It is important to note that these RT-PCR investigations do not represent an exhaustive study of *CLDN *gene expression, but rather a survey of expression in a large number of different human tissues. Follow-up studies on multiple samples for these different malignancies will be necessary to clearly establish the extent and levels of expression of claudins in these tissues. However, it is important to note that the patterns of gene expression obtained by real-time RT-PCR (Figure 3) closely mirrors the *in silico *findings using the SAGE Genie database (Figure 2). For example, the *CLDN3 *and *CLDN4 *expression patterns are consistent between the two analyses (as described above). In addition, we also observe high correspondence in the two approaches when examining *CLDN5 *expression, which appears to be especially high in normal brain and brain cancer. Interestingly, when clustering our RT-PCR data for gene expression patterns, we find *CLDN3,4,7 *are very similar in their expression, suggesting coordinate regulation. The fact that the *CLDN3,4,7 *cluster is present in both normal and tumors suggests that the mechanisms that lead to the coordinated expression of claudins in normal cells is conserved in tumor cells, although it may be inappropriately activated in cancer. It will certainly be interesting to elucidate the mechanisms that lead to the inappropriate activation of these genes.

Our *in silico *and RT-PCR results are consistent with numerous previous reports showing that *CLDN3 *and *CLDN4 *are overexpressed in breast [11], ovarian [7], and prostate tumors [9]. In addition, our data showing overexpression of *CLDN4 *in pancreatic cancer is also in agreement with previous reports [8,27]. The finding of expression of these claudins in other tumors, such as bladder, thyroid, fallopian tubes, stomach, colon, and uterus, is novel and warrants further investigation. CPE-based therapy, which specifically targets cells expressing claudin-3 or claudin-4 [8,9,11,20], may be worth exploring in these malignancies as well. The fact that *CLDN3*, and *CLDN4 *are expressed in several normal tissues (Figure 3A) certainly suggests that systemic administration of CPE may have significant toxic effects. However, the therapeutic index of this compound will depend on the level of up-regulation in the various tumors under study and the mode of administration. In ovarian cancer, for example, where both *CLDN3 *and *CLDN4 *are highly up regulated and where intraperitoneal therapy is possible, CPE treatment is certainly an interesting possibility.

In this report we study the expression of the *CLDN *genes at the mRNA level, but it will obviously be essential to validate these findings at the protein level when all the antibodies are available, as posttranslational mechanisms have been shown to regulate claudin protein levels and localization [5]. In addition, it will be important to investigate the various claudins studied here for their potential clinical use in cancer therapy and diagnosis. With over 20 known members, many of which, as we show in this report, exhibit high tissue-specific expression and deregulation in various cancers, the claudin family of membrane proteins may represent ideal targets for cancer diagnosis and therapy.

## Conclusion

Systematic analysis of CLDN gene expression using *in silico *and RT-PCR approaches demonstrate a wide range of expression patterns among the various claudins in human cancer. *CLDN3*, *CLDN4*, and *CLDN7 *are elevated in several malignancies such as those originating from the pancreas, bladder, thyroid, fallopian tubes, ovary, stomach, colon, breast, uterus, and the prostate. These cancers are thus ideal candidates for a for a novel therapy being developed based on CPE, a toxin that specifically binds claudin-3 and claudin-4. *CLDN18 *is specifically expressed in gastric cells and may represent a marker for gastric tumors. *CLDN5 *is highly expressed in vascular endothelial cells, providing a possible target for antiangiogenic therapy. Overall, a better knowledge of claudin expression in normal and neoplastic tissues may provide new opportunities for the detection, prognosis and therapy of several human cancers.

## Abbreviations

TJ: tight junction; CPE, *Clostridium perfringens *enterotoxin; RT-PCR, reverse-transcriptase PCR; SAGE: serial analysis of gene expression.

## Competing interests

The author(s) declare that they have no competing interests.

## Authors' contributions

KJH: Performed RT-PCR experiments, multiple alignments, and drafted a first draft of the manuscript; RA: Designed and tested some of the primers for the RT-PCR experiments; PJM: Designed and coordinated the study, interpreted the data, and drafted the manuscript.

## Pre-publication history

The pre-publication history for this paper can be accessed here:
